# Absence Seizure Detection Algorithm for Portable EEG Devices

**DOI:** 10.3389/fneur.2021.685814

**Published:** 2021-06-29

**Authors:** Pawel Glaba, Miroslaw Latka, Małgorzata J. Krause, Sławomir Kroczka, Marta Kuryło, Magdalena Kaczorowska-Frontczak, Wojciech Walas, Wojciech Jernajczyk, Tadeusz Sebzda, Bruce J. West

**Affiliations:** ^1^Department of Biomedical Engineering, Wroclaw University of Science and Technology, Wroclaw, Poland; ^2^Department of Pediatric Neurology, T. Marciniak Hospital, Wrocław, Poland; ^3^Department of Child Neurology, Jagiellonian University Medical College, Krakow, Poland; ^4^The Children's Memorial Health Institute, Warszawa, Poland; ^5^Paediatric and Neonatal Intensive Care Unit, Institute of Medical Sciences, University of Opole, Opole, Poland; ^6^Clinical Neurophysiology, Institute of Psychiatry and Neurology, Warszawa, Poland; ^7^Department of Pathophysiology, Wroclaw Medical University, Wroclaw, Poland; ^8^Office of the Director, Army Research Office, Research Triangle Park, Durham, NC, United States

**Keywords:** childhood absence epilepsy, EEG, wavelets, detector, portable device

## Abstract

Absence seizures are generalized nonmotor epileptic seizures with abrupt onset and termination. Transient impairment of consciousness and spike-slow wave discharges (SWDs) in EEG are their characteristic manifestations. This type of seizure is severe in two common pediatric syndromes: childhood (CAE) and juvenile (JAE) absence epilepsy. The appearance of low-cost, portable EEG devices has paved the way for long-term, remote monitoring of CAE and JAE patients. The potential benefits of this kind of monitoring include facilitating diagnosis, personalized drug titration, and determining the duration of pharmacotherapy. Herein, we present a novel absence detection algorithm based on the properties of the complex Morlet continuous wavelet transform of SWDs. We used a dataset containing EEGs from 64 patients (37 h of recordings with almost 400 seizures) and 30 age and sex-matched controls (9 h of recordings) for development and testing. For seizures lasting longer than 2 s, the detector, which analyzed two bipolar EEG channels (Fp1-T3 and Fp2-T4), achieved a sensitivity of 97.6% with 0.7/h detection rate. In the patients, all false detections were associated with epileptiform discharges, which did not yield clinical manifestations. When the duration threshold was raised to 3 s, the false detection rate fell to 0.5/h. The overlap of automatically detected seizures with the actual seizures was equal to ~96%. For EEG recordings sampled at 250 Hz, the one-channel processing speed for midrange smartphones running Android 10 (about 0.2 s per 1 min of EEG) was high enough for real-time seizure detection.

## 1. Introduction

Typical absence seizures are brief (lasting seconds) generalized nonmotor epileptic seizures with an abrupt onset and termination (1, 2). Transient impairment of consciousness and spike-slow wave discharges (SWDs) in electroencephalogram (EEG) are their characteristic manifestations. Typical absence seizures are severe in childhood (CAE) and juvenile (JAE) absence epilepsies but mild or inconspicuous in other syndromes such as juvenile myoclonic epilepsy (JME). Typical absence seizures are predominantly spontaneous, but in about 90% of untreated patients, they may be provoked by hyperventilation. Sleep deprivation, photostimulation, specific geometric patterns, video games, and even thinking may also precipitate them. The pathophysiology of absence seizures is fundamentally different from other types of seizures, making their diagnosis and treatment unique.

CAE is the most common pediatric epileptic syndrome with an age of onset of around 6–8 years (3). It has a prevalence of 10–15% in childhood epilepsies. In children under the age of 16 years, the incidence rate is 1.3 to 6 per 100,000. The ictal EEG of a CAE seizure demonstrates rhythmic 3 Hz bilateral, synchronous, and symmetrical spike and wave discharges (SWDs) with a median duration of approximately 10 s, which on average appear several times per day. In pyknoleptic cases, hundreds of seizures may occur daily (4). The 2010 Childhood Absence Epilepsy Study showed that only 37% of all enrolled subjects were free from treatment failure on their first medication a year after diagnosis (5).

JAE typically begins between 10 and 16 years of age and is usually a life-long condition. JAE seizures tend to be longer than in CAE (lasting up to 45 s) and non-pyknoleptic (typically occurring less than daily).

While CAE and JAE are distinct epilepsy syndromes, there is considerable overlap between them, and the cut-off age remains controversial. During disease, patients with JAE or patients in the overlap group are more likely to develop generalized tonic-clonic seizures and myoclonic attacks. In the long-term follow-up (mean 26 years, range 3–69), only 58% of the patients with absence seizures were in remission (6).

The diagnosis of absence seizures is laborious since it requires analysis of long video-EEGs (on average around 30 minutes long) to detect seizures and their clinical manifestations (consciousness impairment, motor symptoms) and abnormal EEG background activity.

The appearance of low-cost, portable EEG devices (7) has paved the way for long-term, remote monitoring of patients with absence seizures. The potential benefits of this kind of monitoring include facilitation of diagnosis, personalized drug titration, and determining of duration of pharmacotherapy. The need for automatic and reliable detection of absence seizures has long been recognized (8). Diverse algorithms have been proposed so far to detect seizures in animal models of epilepsy (9–12) or in human EEG (13–21). Herein, we present a novel approach to absence seizure detection, which is applicable both to clinical EEGs and recordings made with portable EEG devices with a small number of channels. The algorithm's efficiency and robustness to motion artifacts enable its implementation on mobile devices.

## 2. Materials and Methods

### 2.1. EEG Recordings

Wroclaw Medical University's Ethics Committee approved a retrospective analysis of routine anonymized video-EEG recordings of patients (36 with CAE and 28 with JAE) as well as 30 EEGs of age-matched controls. Epilepsy syndrome was established based on history, age at onset, clinical EEG findings, and neuroimaging. EEGs were acquired with Elmiko Digitrack (BRAINTRONICS B.V. ISO-1032CE amplifier) or Grass Comet Plus EEG (AS40-PLUS amplifier) using 200 or 250 Hz sampling frequency. The international 10-20 standard was used to arrange 19 Ag/AgCl electrodes (impedance below 5k℧). Total EEG duration was equal to 37 and 9 h for the patients and controls, respectively.

We assigned patients' EEG to either training or testing datasets. In the first one, there were 34 recordings (22 CAE and 12 JAE) with 199 seizures (6 ± 4 per patient and averaged seizure duration equal to 12 ± 4 s). In the 30 recordings of the testing dataset (15 CAE and 15 JAE), there were 177 absence seizures (6 ± 5 per patient and averaged duration equal to 12 ± 6 s). An experienced neurologist carried out a visual EEG inspection and marked the seizures with a 1 s accuracy.

**Figure 1:**
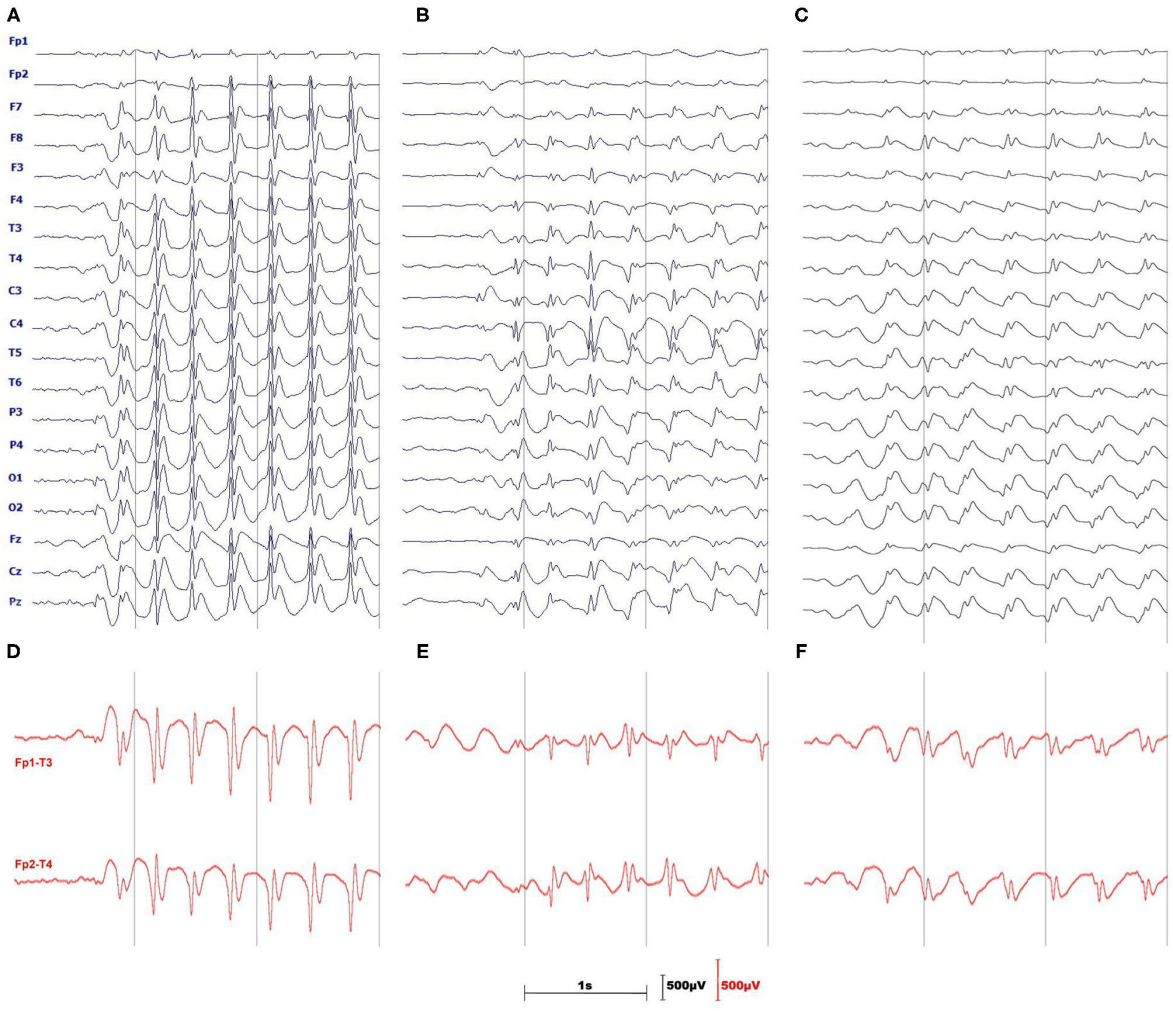
Three monopolar EEGs illustrate the difficulties of absence seizure detection in a single channel. **(A)** is a textbook example of prominent, generalized SWDs. The amplitude of spikes **(B)** or slow waves **(C)** may be small and SWDs may be pronounced only in a handful of channels. **(D–F)** show the advantages of using the longitudinal, bipolar montage which in most cases augments both spikes and slow waves of SWDs.

Figure 1 provides the rationale for using the longitudinal bipolar montage. The seizure detector was developed and tested for two channels: Fp1-T3 and Fp2-T4.

We used three filters for EEG preprocessing: a second-order infinite impulse response (IIR), 6th-order high-pass Butterworth with a cutoff frequency of 0.5 Hz, and 6th-order low-pass Butterworth with a cutoff frequency of 25 Hz. These filters remove 50 Hz power line noise, EEG baseline drift, and muscle artifacts, respectively.

### 2.2. Continuous Wavelet Transform

The continuous wavelet transform (CWT) of a signal *s*(*t*) is an integral transform:

(1) $$\begin{array}{r} T\left[s\right]\left(a,t_{0}\right)=\frac{1}{\sqrt{a}}\int_{-\infty }^{+\infty }s\left(t\right)\psi^{*}\left(\frac{t-t_{0}}{a}\right)dt \end{array}$$

with the basis functions ψ(*a, t*_0_) = ψ(*t*−*t*_0_/*a*), known as wavelets, that are translated and scaled version of the mother function ψ(*t*) (22). Motivated by the results of the previous study (23), as a mother function, we use the complex Morlet wavelet (24, 25):

(2) $$\begin{array}{r} \psi \left(t\right)=\frac{1}{\pi^{1/4}}e^{2\pi if_{c}t}e^{-t^{2}/2} \end{array}$$

whose Fourier transform $\hat{\psi }\left(f\right)$ is given by

(3) $$\begin{array}{r} \hat{\psi }\left(f\right)=\sqrt{2}\sqrt[{4}]{\pi }e^{-2\pi^{2}{\left(f-fc\right)}^{2}}. \end{array}$$

The real parameter *f*_*c*_ is called the center frequency since it is equal to the maximum point of the wavelet's Fourier power spectrum. The scale *a* corresponds to the following pseudofrequency:

(4) $$\begin{array}{r} f_{a}=\frac{f_{c}}{a}. \end{array}$$

As we can see in Equations (2, 3), the wavelets are localized both in time and frequency domains. This dual localization makes CWT particularly applicable to the detection of transient events such as absence seizures.

Seizure detection is based on the properties of the instantaneous wavelet power $|T\left[s\right]\left(a,t_{0}\right){|}^{2}$ normalized by signal's variance σ^2^:

(5) $$\begin{array}{r} w^{\left(n\right)}\left(f_{a},t_{0}\right)=|T\left[s\right]\left(a,t_{0}\right){|}^{2}/\sigma^{2}. \end{array}$$

If we apply the convolution theorem to Equation (1), then it is apparent that the Fourier transform of *T*[*s*](*a, t*_0_) is the pointwise product of the Fourier transforms of the signal and wavelet. Thus, it is possible to calculate CWT by taking the inverse Fourier transform of such a product. We used this approach in the MATLAB function, presented in Supplementary Materials, which calculates the complex Morlet CWT (25). We included the listing to facilitate the reproduction of the results and avoid confusion related to erroneous normalization of the most popular Python and MATLAB CWT implementations. We will discuss this problem in a forthcoming publication.

For the most commonly used wavelets, such as the complex Morlet, the analytical expression for their Fourier transform is known. Therefore, in the presented function, we calculate only the FFT of the signal and use Equation (3) to obtain the wavelet's FFT spectrum.

The complex Morlet CWT of the preprocessed Fp1-T3 and Fp2-T4 channels was calculated without signal partitioning.

### 2.3. Detection Algorithm

**Figure 2:**
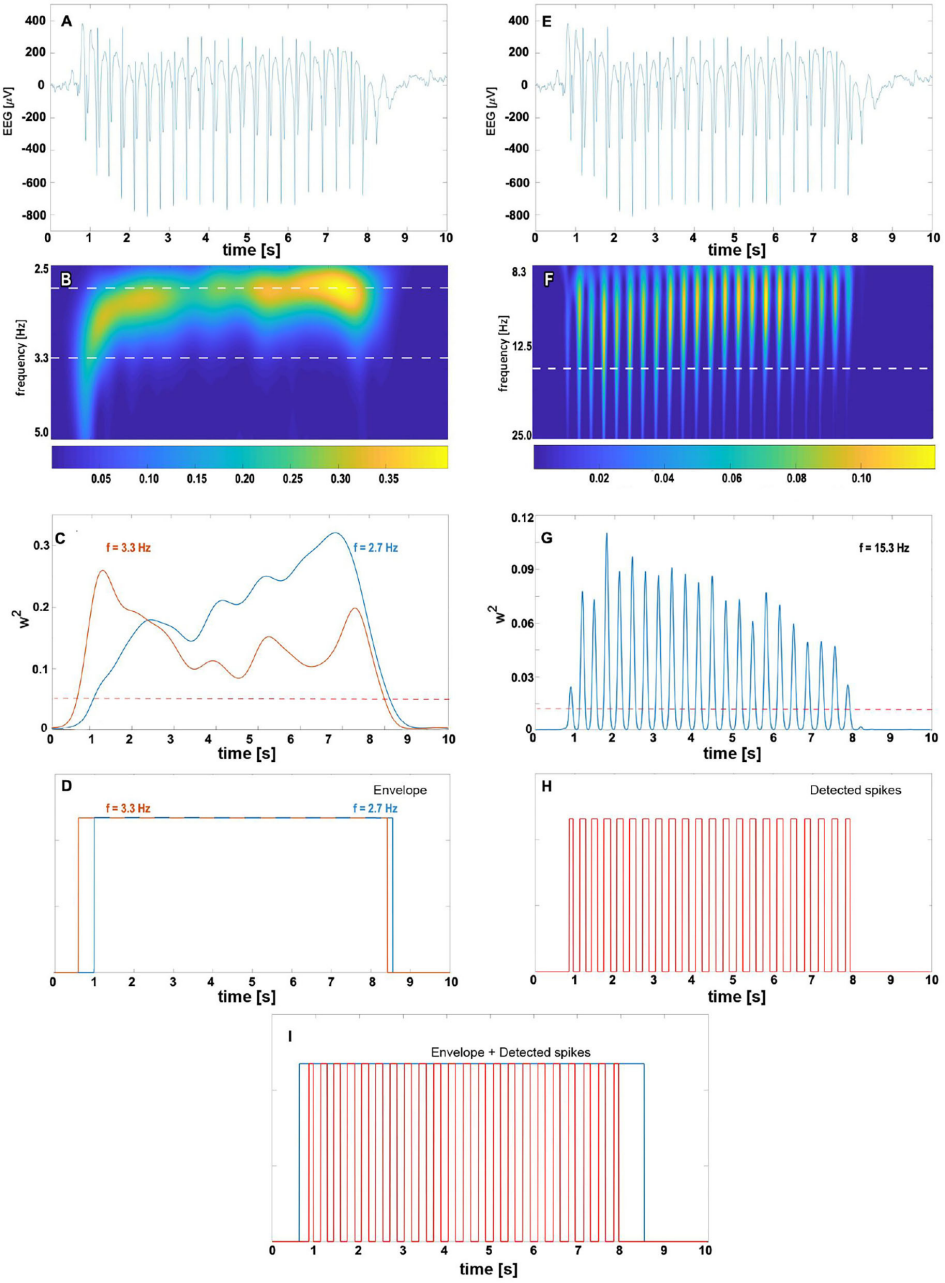
The complex Morlet wavelet analysis of absence slow wave **(B–D)** and spikes **(F–H)**. For clarity, absence EEG is presented at the top of both columns (subplots **A,E**). The density map **(B)** shows the time evolution of normalized wavelet power for pseudo-frequencies in [2.5, 5] Hz range. The 2.7 and 3.3 Hz cuts (marked with the white horizontal dashed line) are plotted in subplot **(C)** with the blue and orange lines, respectively. We refer to time intervals during which the wavelet power for these frequencies exceeds the predetermined threshold (represented in **(C)** by the red dashed horizontal line) as the slow-wave envelopes. For a given seizure, the total envelope is obtained by merging 2.7 and 3.3 Hz envelopes as shown in **(D)**. The right column shows the complex Morlet analysis with parameters tuned to spike detection. The prominent ridges in wavelet power density map **(F)** and peaks in 15.3 Hz cut **(G)** are manifestations of seizure's spikes. The white horizontal dashed line in **(F)** corresponds to the spike frequency 15.3 Hz obtained in the grid search. The train of unit pulses in **(H)** indicates time intervals during which wavelet power for 15.3 Hz is greater than the spike threshold value (marked in subplot **(G)** with the red dashed horizontal line). An absence is detected whenever the epileptic spikes are found in the slow-wave envelope **(I)**.

The detection of an absence seizure (Figures 2A,E), defined as an SWD lasting for more than 2 s (26), proceeds in two steps. First, we locate the train of slow waves and then verify that there are epileptic spikes embedded in it. One can see in the scalogram Figure 2B that when the wavelet's pseudofrequency is close to that of an absence (~3 Hz), then the wavelet power forms a prominent ridge. We refer to the time interval during which the power exceeds the chosen threshold *T*_*E*_ as the slow-wave envelope. This envelope is a unit boxcar function that takes on one whenever the power is greater than *T*_*E*_. As the frequency of SWDs is subject-dependent and may even slightly vary during a seizure (15), we construct two envelopes with wavelet frequencies *f*_*low*_ and *f*_*high*_ (Figure 2C) and merge them as shown in Figure 2D. The merging amounts to a pointwise application of a logical OR function to both envelopes.

For a suitably chosen pseudofrequency *f*_*spike*_, the wavelet power $w^{\left(n\right)}\left(f_{spike}\right)$ peaks around the position of epileptic spikes (Figure 2F). If the percentage of samples *PT* within the final envelope for which the wavelet power is greater than *T*_*S*_, we conclude that there are spikes (Figure 2H) within the envelope (Figure 2I). Such the envelope delineates the absence seizure.

In some cases, $w^{\left(n\right)}\left(f_{spike}\right)$ may also be elevated for high-amplitude artifacts. To reduce the number of false positives, we modified the original algorithm. We do the following amplitude check and disregard all envelopes for which:

- More than 10% of the samples have amplitudes outside the range [−500 μV, 500 μV] (in the differential montage epileptic spikes can have amplitudes of the order of hundreds μV).
- Any sample is outside the range [–1,000 μV, 1,000 μV].

For envelopes shorter than 5 s, we also calculate the variance of $w^{\left(n\right)}\left(f_{spike}\right)$ to detect the wavelet power pulsatility of absence (Figure 2G). If such variance is greater than *T*_*V*_, the detector flags the envelope as a seizure. We refer to such a comparison as the wavelet variance check.

**Figure 3:**
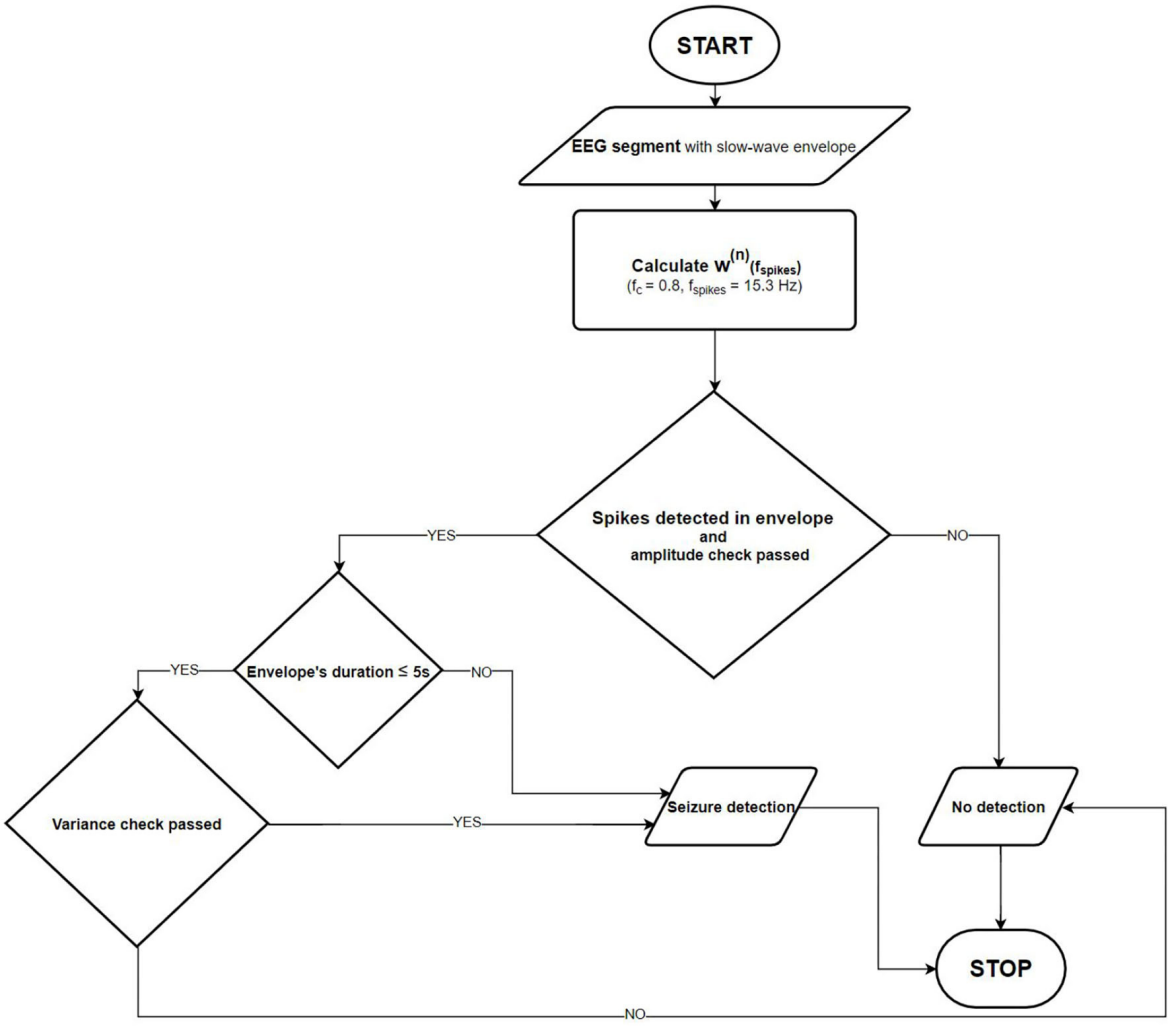
Absence seizure detection flowchart. Once the slow-wave envelope is present in an analyzed EEG segment, the detector checks whether there are epileptic spikes embedded in it. The amplitude and the normalized wavelet power variance checks are also performed to eliminate artifacts.

Figure 3 shows the flowchart of the final absence seizure detection algorithm. The proposed algorithm may be used independently for channels Fp1-T3 and Fp2-T4. Alternatively, the seizure envelopes from these two channels may be superposed.

### 2.4. Determination of Algorithms' Parameters

We determine *f*_*low*_, *f*_*high*_, and *T*_*E*_ by maximizing the overlap of slow-wave envelopes with the absence seizures from a training dataset disregarding possible false detections.

Using these values, we search for the maximum of the following objective function:

(6) $$\begin{array}{r} O\left(f_{spike},T_{S},PT\right)=OVR_{f}-PERR-0.5\times FDET-FDET_{C} \end{array}$$

to find *f*_*spike*_, *T*_*S*_, and *PT*_*S*_ – spike detection parameters. In Eq (6), *OVR*_*f*_ is the percentage overlap of slow-wave envelopes with seizures. *PERR* is the percentage of the number of false positive samples in a given EEG. *FDET* and *FDET*_*C*_ are the number of false detections for the patients and controls, respectively.

The form of the objective function follows two requirements. The first is that we want to overlap the slow-wave envelope with the seizure as accurately as possible. The second is that in patients, false positives can be associated with epileptiform discharges with no clinical manifestations. Therefore, in Eq (6), the weight assigned to the patients' false detection penalty (*FDET*) is half of that given to the controls. We arbitrarily chose the 1:2 weight ratio.

*T*_*V*_ can be determined in the following way. We calculate the variance of $w^{\left(n\right)}\left(f_{spike}\right)$ for the controls' EEGs. Then, we calculate the mean and standard deviation of the distribution. Finally, *T*_*V*_ is set to the mean increased by three standard deviations (*T*_*V*_ = 0.05).

We determined the slow-wave and spike detection parameters using the exhaustive grid search.

### 2.5. Software Implementation

The seizure detection software was implemented both in MATLAB (R2018a) and Java. In the latter case, we wrote a desktop version (which can be run on any computer with Java virtual machine) and a mobile version for Android smartphones. In Java software, we used the class FastFourierTransformer from Apache Commons Math Library (version 3.6.1). Testing and performance benchmarking was performed on a desktop PC with AMD Ryzen 7 3700X 8-Core processor running Windows 10 and Samsung S9 mobile phone (4GB of RAM and 2.8 GHz Samsung Exynos 9810 8-Core processor) with Android 10.

## 3. Results

Using two-step (slow-wave envelope and spike detection) optimization on the training dataset, we obtained the following model parameters *f*_*low*_ = 2.7 Hz, *f*_*high*_ = 3.3 Hz, *T*_*E*_ = 0.05, *f*_*spike*_ = 15.3 Hz, *T*_*S*_ = 0.012, *PT*_*S*_ = 12%. After the parameters were determined, we lowered the value of *T*_*V*_ from 0.05 to 0.008. This change is explained in Discussion section.

The seizure detector had 98.5% and 96.6% sensitivity for the training and testing datasets, respectively (see Supplementary Tables 1, 2). The corresponding false detection rates were equal to 0.9/h and 0.4/h. The overlap *OVR* of the detected and actual seizures was good for both datasets (97% ± 6% and 95% ± 10%). The percentage error *PERR* that accounts for both false positives and erroneously extended slow-wave envelopes was equal to 0.9% ± 0.7% for both datasets.

Supplementary Table 3 shows that both the amplitude and wavelet variance checks contribute to the false detection reduction.

In Supplementary Table 4, we compare the execution times of three absence seizure detector implementations. The execution time is determined by the efficiency of an FFT function, which is used to calculate the continuous wavelet transform. Matlab is renowned for its FFT implementation. Thus, it is not surprising that for the longest segment (*N* = 2^18^), the Matlab version of the detector ran almost 16 and 19 times faster than the Java software running on Windows 10 and Android 10 (0.18 s vs. 2.79 s and 3.41 s). Interestingly enough, for shorter segments, the detector ran faster on the mid-range Android device than on the PC. Nevertheless, the single-channel Android processing speed of 0.2 s per minute of EEG is adequate for real-time seizure detection.

## 4. Discussion

It has long been recognized that long-term EEG monitoring is the most reliable method for absence detection (27). Parents notice only about 6% of daytime seizures, and very often, teachers are the ones who recognize the CAE/JEA beginning (28). The 2010 Childhood Absence Epilepsy Study (5) has provided a compelling rationale for using portable EEG devices in the management of CAE/JEA patients. This randomized controlled trial showed that only 37% of all enrolled subjects were free from treatment failure on their first medication a year after diagnosis.

In the last two decades, many researchers have investigated absence seizure detection (13–17, 20). The datasets in these studies were small–the analyzed SWDs came from nine patients (range 2–20). On average, there were 70 seizures longer than 2 s (range 2–158). In all but one algorithm (20), discrete wavelet transform was used for signal preprocessing. Machine learning was used in 3 of 4 detectors. On average, 11 features were extracted from 15 EEG channels.

Kjaer et al. (19) used an experimental EEG setup with 3 electrodes for 24-h EEG monitoring of 6 patients (593 seizures). Their support vector machine detected 98.4% SWD's with 0.23/h false detection rate using 10 features of five-level db4 wavelet EEG decomposition.

The absence seizure detection algorithm presented in this work is unique because it exploits the apparent traits of SWDs and EEG motion artifacts. Despite the simplicity, its 97.6% accuracy matches that of black-box machine learning classifiers.

**Figure 4:**
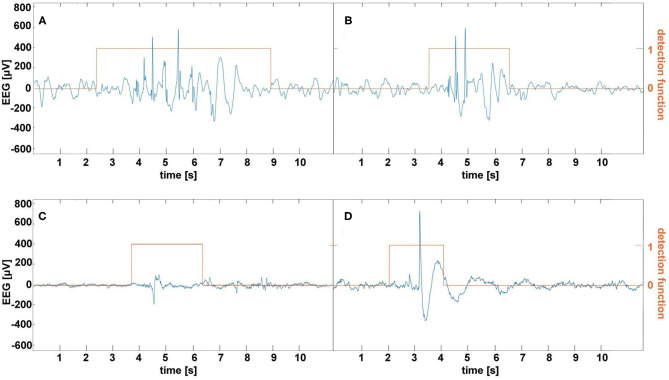
Examples of false absence seizure detection in the EEG of the patients **(A,B)** and controls **(C,D)**. The epileptiform discharges in **(A,B)** were not accompanied by the clinical manifestations. **(C)** shows a rare example of a muscle artifact classified as an absence. A prominent spike-and-wave in **(D)** appeared in a healthy subject's EEG.

Our experience indicates that frequent, albeit not excessively long, EEG home monitoring is feasible in pediatric patients as long as an EEG wearable is easy to put on and is comfortable. This study used the bipolar channels Fp1-T3 and Fp2-T4 for seizure detection because they approximately corresponded to the Muse headband electrode placement. On the one hand, this choice seems to be rational given absence seizures are usually well pronounced in the frontal regions (29) and the large spacing between the electrodes augments the characteristic features of SWDs as shown in Figure 1. On the other hand, Fp1 and Fp2 channels are prone to muscle and eyeblink artifacts. There were 26 and 8 false detections in the patients and controls, respectively. In the patients, all false detections were associated with epileptiform discharges, which did not yield clinical manifestations. Half of the errors in the control group were caused by the prominent SWDs. We show the examples of misclassified EEG segments in Figure 4. We used the stringent value *T*_*V*_ = 0.05 for the determination of the model parameters. Once we realized that false detections are not caused by motion artifacts, for classification, we lowered this parameter to 0.008 to maximize the detection sensitivity (for *T*_*V*_ = 0.05, the sensitivity was equal to 93% with the false detection rate 0.4/h). Some pediatricians agree that sensitivity of 90% and false detection rate of 1/h are clinically acceptable (19).

Unlike previous studies, the false detection rate was not determined by the motion artifacts. We would like to emphasize that the presented seizure detection was performed on clinical EEGs, which is the main limitation of this study. The question arises as to whether the amplitude and wavelet variance checks would be equally effective in eliminating motion artifacts in EEGs acquired with wearable devices in home settings. It is worth mentioning that the false detection rate can be reduced by using secondary electrodes for artifact cancellation (30) and employing different single-channel artifact detectors (31, 32). As most commercial EEG bands have MEMS accelerometers, one may also explore the possibility of incorporating head acceleration in the artifact removal algorithm. However, the connection between EEG artifacts and head movement is not always apparent (33).

In a recent study, Dan et al. presented an absence seizure detector based on a linear multichannel filter that was precomputed offline in a data-driven fashion based on the spatial-temporal signature of the seizure and peak interference statistics (21). The performance of this detector depends on the number of channels (from 3 to 18) used in the calculations. For the three channels, the accuracy was equal to 95% with a 0.4/h false detection rate. The authors set the minimum seizure length to 3 s (34). It is worth pointing out that for this absence duration threshold, the two-channel detector described in this work had the false detection rate equal to 0.5/h (18 and 6 false detections for the patients, and controls, respectively.

To the best of our knowledge, we used the most diverse set of CAE/JAE EEGs (37 h of recordings from 64 patients) for development and testing. The overlap of automatically detected seizures with the actual seizures was high (about 96%). The poor overlap in some patients is predominantly caused by a very small amplitude of the epileptic spikes. Consequently, the detector does not classify such SWD trains as absence seizures. At the end of the seizure, the frequency of SWDs can decrease far below the canonical value of 3 Hz. In this case, the slow-wave envelope is shorter than expected. The frequency drop during the seizure leads to its sfragmentation.

For EEG recordings sampled at 250 Hz, the one-channel processing speed for midrange smartphones running Android 10 was high enough (about 0.2 s per 1 min of EEG) for real-time seizure detection. We found that the detection accuracy was highest for a sliding 30 s EEG buffer, which was shifted by 10 s.

Absence seizure manifestations are mild compared to other epileptic syndromes. Consequently, the rationale for using seizure detectors in CAE/JAE patients is different. Emphasis may be shifted from detection alerts to the facilitation of drug titration and side effects elimination. Unobtrusiveness and ease of use are particularly important for pediatric patients, who may be more willing to tolerate regular EEG measurements if they are incorporated into daily routines such as watching cartoons, playing mobile games, or listening to music.

It is worth pointing out that remote seizure monitoring will be one of the elements of personalized CAE/JAE treatment. There is a growing interest in the development of biomarkers of treatment response and side effects (35). These problems are the subject of our research (36).

## Data Availability Statement

The raw data supporting the conclusions of this article will be made available by the authors, without undue reservation.

## Ethics Statement

The studies involving human participants were reviewed and approved by Wroclaw Medical University's Ethics Committee. Written informed consent for participation was not required for this study in accordance with the national legislation and the institutional requirements.

## Author Contributions

ML, PG, and MK: conceptualization and methodology. PG, ML and MJK: investigation original draft preparation. BW, WW, SK, TS, and WJ: review and editing. All authors contributed to formal analysis.

## Conflict of Interest

The authors declare that the research was conducted in the absence of any commercial or financial relationships that could be construed as a potential conflict of interest.
