# Supplementary material for: Absence Seizure Detection Algorithm for Portable EEG Devices
Source: Front Neurol. 2021 Jun 29;12:685814. doi: 10.3389/fneur.2021.685814 (PMC8275922; doi:10.3389/fneur.2021.685814)
Supplement: Supplementary file 1 [file Data_Sheet_1.PDF]

Supplementary Material

1 SUPPLEMENTARY DATA

Supplementary Listing L1. Matlab function that calculates the Complex Morlet continuous wavelet transform.

```
function [cwtm]=cmorletCWT(signal,avec,dt,fc)
% calculates complex Morlet continuous wavelet transform (cwt)
%
% signal is the input vector
% avec is the vector filled with the scales for which cwt is calculated
% dt is sampling period of the signal
% fc is the center frequency of the complex Morlet wavelet

   N=length(signal);
   Nas=length(avec);

   %%FFT of signal
   dfF=1/(N*dt);
   fftx=fft(signal);
   ff=(-(length(fftx)/2):(length(fftx)/2-1))*dfF;
   waveletMatrix = zeros(Nas,numel(ff));
   fcon=(pi^0.25)*(2^0.5);

   %%FFT of wavelet
   for i=1:Nas
      fftWavelet=sqrt(avec(i))*fcon*exp(-0.5*(((2*pi)*((avec(i)*ff-fc))).^2));
      waveletMatrix(i,:)=ifftshift(fftWavelet);
   end
   fftSigMatrix=repmat(fftx,Nas,1);
   combinedMatrix=fftSigMatrix.*waveletMatrix;
   cwtm=ifft(combinedMatrix,[],2);
end
```

Table S1. Seizure detection performance for the learning dataset. The absence characteristics are shown in the first four columns. The column labels are as follows: EEGD (EEG duration), NABS (numbers of absences), ADABS (average duration of absence), DET (number of detected seizures – true positives), OVR (overlap of the detected seizures with the actual ones), FDET (number of false detections – false positives), PERR (the percentage of false positive samples in a given EEG).

ID [-]	EEGD [s]	NABS [-]	ADABS [s]	DET [-]	OVR [%]	FDET [-]	PERR [%]
1 (CAE)	1800	6	10.50	6	100.00	0	0.51
2 (CAE)	1800	5	13.20	5	100.00	0	1.01
3 (CAE)	1920	10	10.33	10	99.22	1	2.07
4 (CAE)	3000	8	15.75	8	86.23	0	0.96
5 (CAE)	1800	11	11.00	11	100.00	0	1.93
6 (CAE)	3600	6	11.33	6	100.00	0	0.56
7 (CAE)	3300	2	13.50	2	100.00	0	0.15
8 (CAE)	1200	3	15.67	3	100.00	0	0.82
9 (CAE)	3600	7	15.00	7	98.90	1	0.54
10 (CAE)	2100	5	5.33	5	99.91	0	0.56
11 (CAE)	2400	5	8.75	5	98.91	1	0.84
12 (CAE)	2460	10	12.22	10	100.00	0	1.11
13 (CAE)	2100	3	14.33	3	100.00	1	0.43
14 (CAE)	730	2	7.67	2	100.00	0	0.39
15 (CAE)	2100	9	14.11	9	94.99	0	0.39
16 (CAE)	1020	1	5.00	1	100.00	0	0.23
17 (CAE)	1560	3	16.00	3	100.00	1	0.63
18 (CAE)	1200	5	23.20	5	99.94	0	1.20
19 (CAE)	1500	7	9.29	7	100.00	4	2.24
20 (CAE)	1800	2	4.33	2	90.62	2	0.76
21 (CAE)	1010	2	12.50	2	100.00	0	0.54
22 (CAE)	1240	7	16.86	7	99.88	0	1.48
23 (JAE)	1510	2	9.00	2	100.00	0	0.38
24 (JAE)	3200	6	14.19	6	70.23	5	1.52
25 (JAE)	1510	2	9.70	2	100.00	0	0.25
26 (JAE)	1802	3	13.30	3	100.00	0	0.16
27 (JAE)	2402	10	11.21	9	81.93	1	0.64
28 (JAE)	3380	8	13.43	8	100.00	2	1.08
29 (JAE)	3540	11	16.70	11	99.44	0	1.33
30 (JAE)	3000	8	12.44	8	100.00	0	1.13
31 (JAE)	3600	5	12.18	4	91.83	0	0.29
32 (JAE)	1800	17	14.02	16	95.73	0	3.46
33 (JAE)	3780	4	9.80	4	100.00	0	0.82
34 (JAE)	3660	4	14.25	4	100.00	0	0.22

Table S2. Seizure detection performance for the testing dataset. The absence characteristics are shown in the first four columns. The column labels are as follows: EEGD (EEG duration), NABS (numbers of absences), ADABS (average duration of absence), DET (number of detected seizure – true positives), OVR (overlap of the detected seizures with the actual ones), FDET (number of false detections – false positives), PERR (the percentage of false positive samples in a given EEG).

ID [-]	EEGD [s]	NABS [-]	ADABS [s]	DET [-]	OVR [%]	FDET [-]	PERR [%]
35 (CAE)	2700	4	12.25	3	68.09	0	0.12
36 (CAE)	3600	8	14.75	8	85.56	0	0.12
37 (CAE)	2700	14	11.14	14	99.66	1	1.00
38 (CAE)	2100	12	13.56	12	99.92	1	1.77
39 (CAE)	1020	3	19.67	3	100.00	0	0.55
40 (CAE)	1920	7	9.29	7	99.81	0	1.08
41 (CAE)	3640	3	11.00	3	100.00	1	0.94
42 (CAE)	1515	5	12.80	5	100.00	0	0.93
43 (CAE)	3010	16	6.36	14	92.91	2	1.07
44 (CAE)	2280	4	8.80	4	100.00	0	0.30
45 (CAE)	1970	12	7.00	12	90.72	0	1.94
46 (CAE)	2460	11	17.20	9	94.99	0	0.44
47 (CAE)	1360	2	13.50	2	100.00	0	0.87
48 (CAE)	1330	3	18.86	3	99.32	0	0.91
49 (CAE)	1300	6	6.80	6	98.39	0	2.30
50 (JAE)	1260	3	10.47	3	100.00	0	0.90
51 (JAE)	2050	1	8.50	1	100.00	1	0.27
52 (JAE)	920	1	8.70	1	100.00	0	0.12
53 (JAE)	1130	1	5.00	1	100.00	0	0.15
54 (JAE)	1290	4	8.22	4	100.00	0	0.77
55 (JAE)	1260	6	6.58	5	85.27	0	0.87
56 (JAE)	2100	2	10.70	2	99.48	0	0.15
57 (JAE)	2225	6	17.10	6	100.00	0	0.81
58 (JAE)	1200	7	13.80	7	84.36	0	3.05
59 (JAE)	1861	18	3.75	18	83.33	0	1.16
60 (JAE)	2340	4	13.25	4	100.00	0	1.08
61 (JAE)	2520	1	7.00	1	100.00	0	0.03
62 (JAE)	1460	3	35.67	3	55.99	0	1.96
63 (JAE)	1370	5	13.60	5	100.00	0	0.79
64 (JAE)	1360	5	8.93	5	99.17	1	0.82

Table S3. The amplitude and wavelet variance checks' contributions to false detection reduction. We present the number of false detections (in the learning and testing datasets) with the checks turned on (+) or off (-).

	$Fp_1 - T_3$				$Fp_2 - T_4$			
Amplitude check	-	+	-	+	-	+	-	+
Variance check	-	-	+	+	-	-	+	+
False Detections	102	89	53	15	152	105	58	19
PERR [%]	1.02	0.87	0.85	0.71	1.01	0.86	0.80	0.67

Table S4. Execution times for the Matlab and Java implementations of the absence seizure detector. We benchmarked the implementations for three EEG segment lengths. The mean values were calculated using the first 10 EEG recordings of the training dataset (Table S1). For the sampling frequency equal to 250 Hz, the segment length $N = 2^{18}$ corresponds to 17 min of EEG.

	Matlab 2018a Win 10			Java Win 10			Android 10		
N	2^{16}	2^{17}	2^{18}	2^{16}	2^{17}	2^{18}	2^{16}	2^{17}	2^{18}
Mean [s]	0.04	0.11	0.18	2.36	2.50	2.79	0.86	1.60	3.41
Std [s]	0.01	0.02	0.02	0.68	0.74	0.62	0.07	0.07	0.12
